# Synergistic and dose-dependent pharmacological effects of black shallot and nano-curcumin combination on nociceptive, pyretic, and inflammatory responses in murine models

**DOI:** 10.5114/bta/215226

**Published:** 2026-03-06

**Authors:** Nhung Thi Phuong Tran, Ngan Thi Nguyen, Quan Hong Bui

**Affiliations:** Institute of Biotechnology and Food Technology, Industrial University of Ho Chi Minh City, Vietnam

**Keywords:** black shallot extract, nano-curcumin, dose-response, antinociceptive, antipyretic, anti-inflammatory

## Abstract

**Background:**

Inflammation, pain, and fever are common features of chronic diseases and are often inadequately controlled by existing therapies. This study evaluated a fixed-ratio combination of black shallot extract and nano-curcumin (EBSC) for dose-dependent and synergistic antinociceptive, antipyretic, and anti-inflammatory effects in murine models.

**Materials and methods:**

EBSC (100, 200, and 300 mg/kg; oral) was assessed in Swiss albino mice using the hot-plate and tail-flick tests (antinociception), Brewer’s yeast-induced pyrexia (antipyretic), and carrageenan-induced paw edema (anti-inflammatory). TRPV1, COX-2, PGE_2_, TNF-α, IL-6, and IL-10 levels were quantified by ELISA. Acute oral toxicity was evaluated according to OECD 423. Data were analyzed using one-way analysis of variance (ANOVA) with Tukey post hoc tests; dose-response relationships were modeled using linear and Hill fits.

**Results:**

EBSC produced dose-dependent analgesic effects: hot-plate reaction latency increased from 37.92 ± 1.24 s (100 mg/kg) to 40.05 ± 1.89 s (300 mg/kg), and pain inhibition increased to 62.48 ± 1.73% (*p* < 0.001). Tail-flick latency also significantly prolonged. EBSC at 300 mg/kg reduced pyrexia by 1.25 ± 0.13°C (*p* < 0.001). Carrageenan-induced paw edema was inhibited by 46.2% at 4 h with EBSC300 (*p* < 0.001). Molecular markers (TRPV1, COX-2, PGE_2_, TNF-α, IL-6) decreased in a dose-dependent manner, whereas IL-10 levels increased. Dose-response models demonstrated strong fits (*R*^2^ > 0.98). No mortality or significant biochemical alterations were observed up to 5.000 mg/kg.

**Conclusions:**

EBSC exhibits dose-dependent and synergistic antinociceptive, antipyretic, and anti-inflammatory activities with a favorable acute safety profile, supporting its potential for further development as a standardized phytopharmaceutical.

## Introduction

Inflammation, pain, and fever are characteristic responses associated with a wide range of acute and chronic diseases, including hormone-sensitive malignancies, and are often driven by oxidative stress and dysregulated immune signaling (Ahmed et al. 2024). Although conventional therapies can be effective in the short term, their use is frequently limited by systemic toxicity, non-specificity, and the development of resistance (Han et al. 2025). These limitations have fueled growing interest in phytopharmaceuticals that offer multi-targeted activity with improved safety profiles (Mardiana et al. 2025).

Black shallot (*Allium ascalonicum*) and nano-curcumin are two phytocompounds with well-established anti-inflammatory, antioxidant, and analgesic properties (Nhung and Quoc 2024; Serini et al. 2025). Bioactive sulfur compounds and flavonoids in black shallot modulate cytokine production and desensitize nociceptive ion channels such as TRPV1 (Nhung 2025), whereas nano-curcumin inhibits COX-2-mediated prostaglandin E_2_ (PGE_2_) synthesis and downregulates TRPV1 activation (Karthikeyan et al. 2020). These molecular targets – TRPV1 (central to nociceptive signaling), COX-2 and PGE_2_ (key mediators of pyretic and inflammatory pathways), TNF-α and IL-6 (pro-inflammatory cytokines), and IL-10 (an anti-inflammatory cytokine) – play essential roles in regulating pain, fever, and inflammation.

Despite the known therapeutic potential of each compound individually, the fixed-ratio combination of black shallot extract and nano-curcumin (EBSC) has not previously been explored with respect to dose-response behavior or synergistic pharmacodynamics. This study evaluates the therapeutic effects and mechanistic relevance of EBSC in murine models of nociception, pyrexia, and inflammation, providing a scientific basis for its advancement as a standardized, multi-targeted phytopharmaceutical.

## Materials and methods

### Extraction and combination of EBSC materials

Fresh purple shallots (*Allium ascalonicum*) were collected from Lý Sơn Island, Vietnam, and converted into black shallots through thermal aging at 70°C and 70% humidity for 21 days (Tran and Tran 2025). Ethanol extraction was performed using 70% EtOH (1 : 10 w/v, 72 h × 3), followed by filtration, vacuum evaporation at 60°C, and freeze-drying. Nano-curcumin (NovaSOL^®^, Germany) was prepared in 0.5% CMC. EBSC was formulated by combining equal weights (1 : 1, w/w) of black shallot extract and nano-curcumin based on preliminary findings indicating complementary bioactivities. A dose range of 100–300 mg/kg was selected to evaluate potential linear and non-linear dose-response correlations.

### Phytochemical profiling and content determination

EBSC was screened for secondary metabolites using standard qualitative methods (Tran and Tran 2021; Moreno-Ortega et al. 2023; Kasetsuwan et al. 2024). Quantitative assays measured total phenolics (Folin-Ciocalteu, mg GAE/g), flavonoids (AlCl_3_ method, mg QE/g), and alkaloids (bromocresol green, mg AE/g) (Tran et al. 2023). Curcuminoid content was quantified spectrophotometrically using a UV-Vis spectrophotometer (Shimadzu UV-1900, Japan) at 425 nm, with curcumin equivalents (mg CE/g) calculated from a standard calibration curve as described by Chumroenphata et al. (2021). All measurements were performed in triplicate.

### LC-MS/MS-based characterization of phytochemicals

Phytochemical constituents were identified using LC-MS/MS with a C18 column, gradient elution (H_2_O + 0.1% FA/ACN + 0.1% FA), and MRM mode. Compounds were identified based on retention time, *m/z* values, and comparison with authentic standards (Abilkassymova et al. 2024).

### Experimental animals and ethical compliance

Male Swiss albino mice (28 ± 2 g) were housed in ventilated cages containing autoclaved rice husk bedding and maintained at 24 ± 2°C, 55–65% humidity, and a 12/12 h light/dark cycle. Animals received ad libitum access to standard rodent chow and filtered water. After a 7-day acclimatization period, experiments commenced. All procedures were approved by the Institutional Animal Ethics Committee and adhered to international guidelines for the care and use of laboratory animals.

Tissue samples (spinal cord, inflamed paw) were collected 4 h after the final treatment under anesthesia and processed immediately for biochemical analysis.

### Acute oral toxicity study

A single oral dose of EBSC (1.000, 3.000, or 5.000 mg/kg) was administered to female Swiss albino mice (*n*= 6/group) via oral gavage using flexible polypropylene tubes, following OECD Guideline 423 (OECD 2022). Clinical signs were recorded as changes in posture, activity, coat condition, locomotion, respiration, and food intake. Body weight and survival were monitored for 14 days. A gross necropsy was performed on day 14. Serum biochemical markers (ALT, AST, CK, creatinine, and WBC count) were assessed to evaluate systemic toxicity.

### Experimental design

Mice (*n* = 8/group) were randomly assigned to the following groups: normal control (distilled water), disease control, vehicle control (0.5% CMC), reference drug, and EBSC treatment (100, 200, or 300 mg/kg). All substances were administered once daily for 3 days via oral gavage. Pyrexia and inflammation were induced using 20% Brewer’s yeast and 1% carrageenan, respectively. Reference drugs included tramadol (10 mg/kg), paracetamol (150 mg/kg), and indomethacin (10 mg/kg).

### Analgesic activity

#### Hot plate test

Mice were individually placed on a hot plate (54 ± 1°C), and the latency to nociceptive response (paw licking or jumping) was recorded at 0, 15, 30, 45, and 60 min posttreatment. A 30-s cut-off was used to avoid tissue damage. Analgesic efficacy was calculated as the percentage of pain inhibition (IPH, %) relative to baseline. All procedures were conducted under blinded conditions (Nhung 2025).

#### Tail flick test

The distal 2–3 cm of the tail was immersed in water maintained at 50 ± 1°C, and the latency to tail withdrawal was recorded at predetermined intervals. A 15-s cut-off was applied to prevent injury. Analgesic activity was expressed as the percentage inhibition of the tail-flick response (IFT, %) relative to baseline. All assessments were performed under blinded conditions (Quan and Nhung 2025).

#### TRPV1 quantification

Spinal cord samples were homogenized in ice-cold PBS and centrifuged at 3,000 rpm for 10 min at 4°C. Supernatants were analyzed using TRPV1-specific ELISA kits (Elabscience^®^), and absorbance was measured at 450 nm. TRPV1 concentrations were expressed as ng/ml and normalized using the Bradford assay. All measurements were conducted in duplicate under standardized conditions (Liu et al. 2024).

### Antipyretic activity

#### Brewer’s yeast-induced pyrexia

Fever was induced by subcutaneous injection of 20% Brewer’s yeast (10 ml/kg). After 18 h, febrile mice (≥ 0.5°C above baseline) received oral administration of the test compounds. Rectal temperatures were measured at 0, 1, 2, and 3 h posttreatment. Antipyretic efficacy was calculated as the percentage reduction in rectal temperature (IPR, %). All assessments were blinded (Zubair et al. 2025).

#### COX-2 and PGE_2_ quantification

Inflamed paw tissues were homogenized in cold PBS, centrifuged at 4°C, and supernatants analyzed using mouse-specific ELISA kits (Elabscience^®^, USA). Absorbance was measured at 450 nm, and concentrations were normalized to total protein (pg/mg). All analyses were conducted in duplicate (Li et al. 2025).

### Anti-inflammatory activity

#### Carrageenan-induced paw edema

Acute inflammation was induced by subplantar injection of 1% carrageenan (50 µl) into the right hind paw. Paw thickness was measured using a digital caliper at 0, 1, 2, 3, and 4 h postinjection. Anti-inflammatory efficacy was expressed as the percentage inhibition of paw edema relative to the negative control. All evaluations were performed under blinded conditions (Fatima et al. 2025).

#### Cytokine profiling

Paw tissues were homogenized in PBS, centrifuged at 4°C, and supernatants analyzed for TNF-α, IL-6, and IL-10 using ELISA kits (Elabscience^®^, USA). Absorbance was recorded at 450 nm, and concentrations were expressed as pg/mg protein. All assays were conducted in duplicate under blinded conditions (Tran et al. 2023).

### Statistical analysis and dose-response modeling

Data are presented as mean ± standard deviation (SD). Statistical comparisons were performed using one-way analysis of variance (ANOVA) followed by Tukey’s post hoc test. Dose-response relationships were evaluated using linear and nonlinear regression models. Correlation strength and model fit were assessed using Pearson’s *r* and *R^2^*. Sigmoidal curves were fitted using the Hill equation, and model accuracy was validated using Akaike information criterion (AIC) and residual diagnostics.

## Results

All data are presented as mean ± SD, with *n*= 8 mice per group. Statistical comparisons were performed using one-way ANOVA followed by Tukey’s post hoc test, with significance accepted at *p*< 0.05.

### Phytochemical composition of EBSC

EBSC exhibited a complex phytochemical profile consisting of polyphenols, flavonoids, alkaloids, saponins, and curcuminoids (Table S1). Quantitatively, the formulation contained 73.41 ± 2.37 mg GAE/g of total polyphenols, 44.78 ± 1.74 mg QE/g of flavonoids, and 29.95 ± 0.38 mg CE/g of curcuminoids. These comparatively high levels align with antioxidant-rich botanicals and suggest that phenolic and curcuminoid fractions likely contribute substantially to EBSC’s redox-modulating and anti-inflammatory properties. The abundance of these secondary metabolites provides a strong chemical rationale for the pharmacological outcomes observed in the subsequent in vivo assays.

### LC-MS/MS profiling

LC-MS/MS analysis identified key bioactive constituents originating from both black shallot and nano-curcumin components (Table S2). Major compounds included gallic acid, cyanidin-3-glucoside, S-allyl cysteine (SAC), S-methyl-L-cysteine sulfoxide, and the curcuminoids (curcumin, desmethoxycurcumin, and bisdemethoxycurcumin). The concurrent presence of organosulfur compounds (e.g., SAC) and curcuminoids highlights EBSC’s multi-target pharmacophore: sulfur derivatives likely contribute antioxidant effects and TRPV1 modulation, whereas curcuminoids predominantly suppress COX-2/PGE_2_ biosynthesis. These compositional data support the anticipated biochemical complementarity of the 1 : 1 formulation.

### Acute oral toxicity and safety profile

No mortality or observable clinical abnormalities (grooming, posture, locomotion, respiration) occurred during the 14-day observation period following single oral doses up to 5.000 mg/kg, indicating a wide safety margin (LD_50_ > 5.000 mg/kg). Serum biochemical parameters (ALT, AST, creatinine, and CK) and hematological indices (WBC) remained within physiological limits and did not differ significantly from the controls (*p* > 0.05). These findings demonstrate that EBSC is well tolerated at pharmacologically relevant doses and support its use in the repeated-dose efficacy studies.

### Analgesic activity

#### Hot plate test

**Figure 1:**
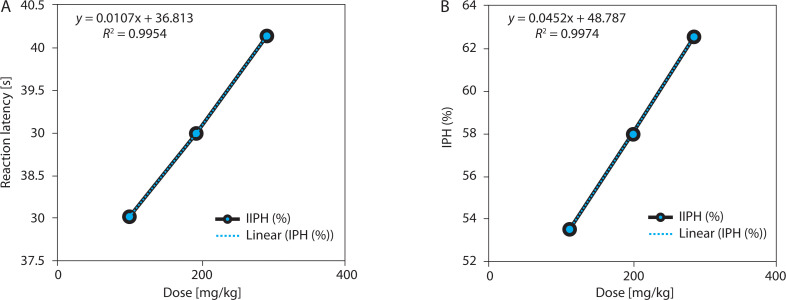
Dose-response relationship between EBSC and hot-plate outcomes. (**A**) Reaction latency (s). (**B**) Pain inhibition (IPH, %). Data are presented as mean ± SD (n = 8 per group). Statistical comparisons were performed using one-way ANOVA followed by Tukey’ s post hoc test. Linear regression assessed the dose-response correlation; R^2^ values are shown in the figure panels

EBSC produced a statistically significant, dose-dependent prolongation of nociceptive latency (one-way ANOVA with Tukey’s post hoc test, *p* < 0.001). Based on the absolute posttreatment latencies (Table 1), reaction time increased from 37.92 ± 1.24 s (EBSC100) to 40.05 ± 1.89 s (EBSC300), representing a 5.6% increase at the highest dose (Table 1 and Figure 1A). Notably, the pain-inhibition index (IPH), which reflects baseline-normalized efficacy, rose markedly to 62.48 ± 1.73% at EBSC300 (Table 1), indicating a substantial functional analgesic effect despite modest changes in absolute seconds. The time-course profile showed a progressive increase in latency from 15 to 60 min with a peak at 60 min. Dose-response regression demonstrated a strong positive relationship between EBSC dose and analgesic effect (*R*^2^ = 0.987; *r* = 0.994; Figure 1B). Values are expressed as mean ± SD (*n* = 8); group differences were assessed by one-way ANOVA with Tukey’s post hoc test.

**Table 1:** Dose-dependent effect of EBSC on reaction latency (RLH) and pain inhibition (IPH) in the hot plate test

Dose [mg/kg]	RLH [s]	IPH (%)	*R* ^2^	*r*	*p*-value	Correlation
100	37.92 ± 1.24	53.44 ± 1.34	0.994	0.989	0.0146	↑↑
200	38.86 ± 1.47	57.56 ± 1.58	0.985	0.996	0.0177	↑↑
300	40.05 ± 1.89	62.48 ± 1.73	0.987	0.994	0.0128	↑↑

Values are expressed as mean ± SD (*n* = 8). Statistical analysis was performed using one-way ANOVA followed by Tukey’s post hoc test. ↑↑ indicates a strong positive correlation between EBSC dose and analgesic outcome. *p* < 0.05 was considered statistically significant.

#### Tail-flick test

**Figure 2:**
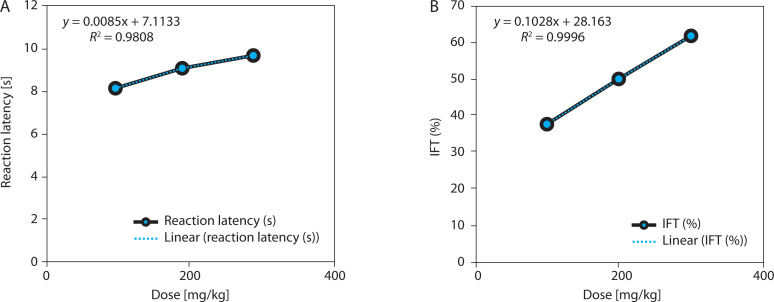
Dose-response relationship in the tail-flick assay. (**A**) Withdrawal latency (s). (**B**) Pain inhibition (IFT, %). Data are presented as mean ± SD (n = 8 per group). Statistical comparisons were performed using one-way ANOVA with Tukey’ s post hoc test. Regression analysis evaluated dose-response relationships; R^2^ values are displayed in the panels

EBSC also produced a statistically significant, dose-dependent increase in tail-withdrawal latency (one-way ANOVA with Tukey’s post hoc test, *p* < 0.001). Based on the absolute posttreatment latencies (Table 2), withdrawal time increased from 7.89 ± 0.36 s (EBSC100) to 9.58 ± 0.55 s (EBSC300), representing a modest absolute increase (21.4%) at the highest dose (Table 2 and Figure 2A). However, the functional analgesic measure, the tail-flick inhibition index (IFT), rose sharply to 58.88 ± 1.79% in the EBSC300 group, indicating a pronounced peripheral antinociceptive effect despite the relatively modest raw latencies. Time-course data showed progressive latency increases across measurement intervals. Dose-response regression demonstrated excellent model fit (*R*^2^ = 0.984–0.993; Figure 2B), confirming robust dose dependence. Data represent mean ± SD (*n* = 8); statistical significance was determined using one-way ANOVA followed by Tukey’s post hoc test (Table S3).

**Table 2:** Effect of EBSC on tail flick reaction latency (RLT) and pain inhibition (IFT) in mice

Dose [mg/kg]	RLT [s]	IFT (%)	*R* ^2^	*r*	*p*-value	Correlation
100	7.89 ± 0.36	38.32 ± 1.67	0.989	0.997	0.0178	↑↑
200	8.94 ± 0.49	48.97 ± 1.43	0.991	0.986	0.0182	↑↑
300	9.58 ± 0.55	58.88 ± 1.79	0.995	0.988	0.0169	↑↑

Data are shown as mean ± SD (*n* = 8). One-way ANOVA with Tukey’s post hoc test was applied. ↑↑ indicates a strong positive dose-response correlation.

#### TRPV1 modulation

EBSC significantly downregulated spinal TRPV1 in a dose-dependent manner (Table S4). TRPV1 levels decreased from 8.98 ± 0.19 ng/ml (hot plate, EBSC100) to 5.65 ± 0.15 ng/ml (EBSC300), and from 9.66 ± 0.22 ng/ml (tail flick, EBSC100) to 5.79 ± 0.17 ng/ml (EBSC300) (*p* < 0.001). These reductions paralleled improvements in nociceptive thresholds, producing a strong inverse correlation between TRPV1 levels and analgesic indices (*r* = –0.981; *R*^2^ = 0.963). These results indicate that EBSC’s antinociceptive action is at least partially mediated through TRPV1 downregulation/desensitization, reducing peripheral nociceptor activation and central pain transmission. Data are expressed as mean ± SD (*n* = 8) and were analyzed by one-way ANOVA with Tukey’s post hoc test.

### Antipyretic activity

#### Brewer’s yeast-induced pyrexia

**Figure 3:**
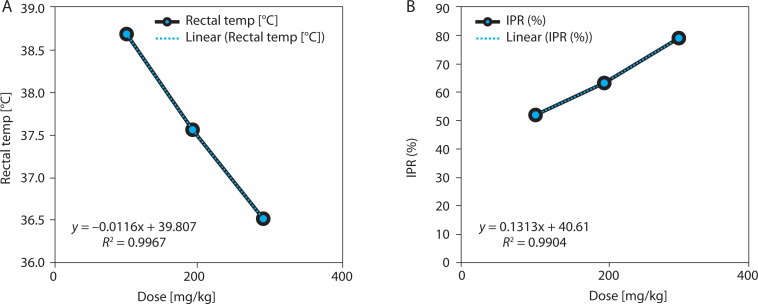
Dose-dependent antipyretic effects of EBSC in Brewer’ s yeast-induced pyrexia. (**A**) Change in rectal temperature (°C). (**B**) Fever inhibition percentage (IPR, %). Data are presented as mean ± SD (n = 8 per group). Statistical comparisons were performed using one-way ANOVA followed by Tukey’ s post hoc test. Regression analysis assessed dose-response correlation (R^2^ values are shown in the figure panels)

EBSC significantly reduced yeast-induced hyperthermia in a clear dose-dependent manner (Table 3 and Figure 3A). At 300 mg/kg, the mean rectal temperature decreased by 1.25 ± 0.13°C, compared with a negligible reduction of 0.26 ± 0.11°C in the negative control group (*p* < 0.001). This temperature reduction corresponded to a fever-inhibition rate of 80.7%, closely approximating the effect of paracetamol (150 mg/kg). Lower doses of EBSC (100 and 200 mg/kg) also produced significant yet progressively smaller reductions, consistent with a graded dose-response pattern. Regression analysis showed a strong positive correlation between EBSC dose and fever inhibition (*R*^2^ = 0.989; Fig. 3B), confirming the reproducibility and predictability of EBSC’s antipyretic response. Data represent mean ± SD (*n* = 8); comparisons among groups were made by one-way ANOVA with Tukey’s post hoc test.

**Table 3:** Dose-dependent antipyretic effect of EBSC in the Brewer’s yeast-induced pyrexia model

Dose [mg/kg]	RT [^o^C]	IPR (%)	*R* ^2^	*r*	*p*-value	Correlation
100	38.69 ± 0.01	54.48 ± 0.55	0.991	0.984	0.0147	↓↓/↑↑
200	37.42 ± 0.03	65.37 ± 0.67	0.987	0.994	0.0152	↓↓/↑↑
300	36.38 ± 0.02	80.73 ± 0.49	0.989	0.996	0.0174	↓↓/↑↑

Rectal temperature (RT) and fever inhibition percentage (IPR) are shown as mean ± SD (*n* = 8). One-way ANOVA with Tukey post hoc test was used. ↑↑ indicates a strong positive correlation with IPR; ↓↓ indicates a strong negative correlation with RT.

#### COX-2 and PGE_2_ suppression

Consistent with its antipyretic action, EBSC induced a clear, dose-dependent suppression of COX-2 and PGE_2_ in inflamed paw tissue (Table S5). COX-2 decreased from 180.7 ± 6.2 pg/mg (model group) to 99.2 ± 5.6 pg/mg (EBSC300), while PGE_2_ decreased from 398.1 ± 10.4 pg/mg to 205.3 ± 8.9 pg/mg (*p* < 0.001). These reductions represent ~45% inhibition for COX-2 and ~48% inhibition for PGE_2_, demonstrating effective EBSC interference with the prostaglandin synthesis pathway responsible for febrile responses. Regression analysis confirmed a strong dose-dependent correlation (*R*^2^ > 0.99), demonstrating consistent biochemical modulation across treatment levels. Collectively, these results provide a mechanistic explanation for the normalization of body temperature observed *in vivo*. All values represent mean ± SD (*n* = 8); statistical analysis was performed by one-way ANOVA with Tukey’s post hoc test.

### Anti-inflammatory activity

#### Carrageenan-induced edema

**Figure 4:**
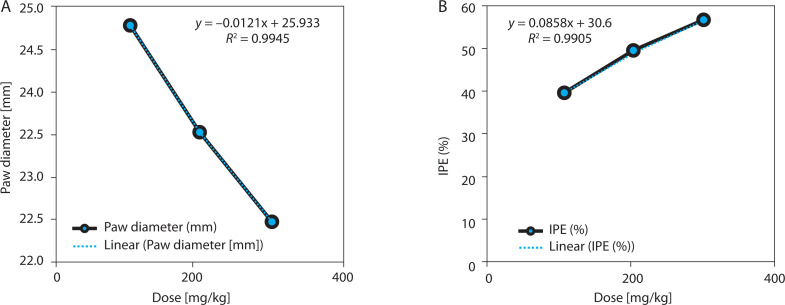
EBSC effects on carrageenan-induced paw edema. (A) Paw thickness (mm). (B) Edema-inhibition percentage (IPE, %). Data are presented as mean ± SD (n = 8 per group). Statistical comparisons were performed using one-way ANOVA with Tukey’ s post hoc test. Linear regression illustrated dose-response trends (R^2^ values are shown in the panels)

EBSC produced a significant, dose-dependent inhibition of carrageenan-induced paw edema (Table 4 and Figure 4A). At 4 h post carrageenan injection, paw thickness decreased from 3.21 ± 0.16 mm (model) to 1.72 ± 0.11 mm (EBSC300) (*p* < 0.001), corresponding to 46.2% inhibition. Lower doses (100 and 200 mg/kg) showed graded decreases in edema, confirming a consistent dose-response effect. The time-course profile indicated that the anti-inflammatory effect began within 1 h and peaked between 3 and 4 h, suggesting suppression of both early (histamine/serotonin-mediated) and late (prostaglandin/cytokine-mediated) inflammatory phases. Regression analysis revealed a strong inverse correlation between EBSC dose and paw thickness (*R*^2^ = 0.987; Figure 4B), supporting a dose-dependent anti-inflammatory mechanism. Values represent mean ± SD (*n* = 8); group differences were evaluated by one-way ANOVA followed by Tukey’s test.

**Table 4:** Dose-dependent inhibition of paw edema by EBSC in the carrageenan-induced inflammation model

Dose [mg/kg]	PT [mm]	IPE (%)	*R* ^2^	*r*	*p*-value	Correlation
100	24.78 ± 0.15	38.69 ± 1.17	0.994	0.992	0.0155	↓↓/↑↑
200	23.42 ± 0.13	48.72 ± 1.19	0.991	0.989	0.0173	↓↓/↑↑
300	22.37 ± 0.16	55.84 ± 1.21	0.987	0.995	0.0147	↓↓/↑↑

Paw thickness (PT) and inhibition percentage (IPE) are expressed as mean ± SD (*n* = 8). One-way ANOVA and Tukey’s test applied. ↑↑ indicates a positive correlation in IPE; ↓↓ indicates a dose-dependent reduction in paw thickness.

#### Cytokine modulation

EBSC shifted cytokine expression toward an anti-inflammatory profile (Table S6). TNF-α decreased from 215.47 ± 1.35 to 168.79 ± 1.34 pg/ml and IL-6 from 46.66 ± 1.17 to 36.28 ± 1.14 pg/ml (one-way ANOVA with Tukey’s post hoc test, *p* < 0.05), each showing strong negative correlations with dose (*r* = –0.99). Conversely, IL-10 increased, from 119.83 ± 2.51 (EBSC100) to 148.77 ± 2.88 pg/ml (EBSC300; *p* < 0.05), exceeding the model value (98.4 ± 5.1 pg/ml). IL-10 exhibited a strong positive correlation with EBSC dose (*r* = 0.99). These coordinated shifts reflect suppression of pro-inflammatory signaling and enhancement of anti-inflammatory responses.

#### Integrated dose-response relationships

Comprehensive regression modeling (Table S7) demonstrated strong and consistent dose-response relationships across all behavioral and molecular parameters (*R^2^* > 0.98; *p* < 0.05). Functional outcomes – including reaction latency, pain inhibition, and fever reduction – increased with EBSC dose, indicating enhanced efficacy at higher concentrations. IL-10 also rose proportionally with dose, suggesting amplified immunoregulatory activity. In contrast, molecular mediators of nociception and inflammation, including TRPV1, COX-2, PGE_2_, TNF-α, and IL-6, demonstrated robust negative correlations with dose, reflecting progressive biochemical suppression as the dosage increased. These integrated findings across behavioral, biochemical, and immunological domains confirm that EBSC exerts predictive, dose-dependent, multi-target pharmacological effects, supporting its therapeutic optimization and translational potential. Results (mean ± SD, *n* = 8) were statistically assessed by one-way ANOVA with Tukey’s post hoc test applied for pairwise group comparisons.

## Discussion

EBSC comprises a fixed-ratio combination of black shallot extract and nano-curcumin, each known for distinct yet complementary pharmacological properties. LC-MS/MS profiling confirmed the co-presence of curcuminoids, organosulfur compounds, flavonoids, and phenolic acids-bioactives with overlapping and additive mechanisms. Curcumin is recognized for inhibiting NF-κB signaling and downregulating pro-inflammatory mediators such as TNF-α, IL-6, and COX-2 (Kunnumakkara et al. 2023). Simultaneously, sulfur-containing compounds from *Allium* species enhance endogenous antioxidant defenses and desensitize nociceptive ion channels, including TRPV1 (Tran et al. 2023a).

The observed analgesic efficacy in the hot-plate and tail-flick models, together with dose-dependent TRPV1 suppression, reflects both central and peripheral modulation. These effects are likely potentiated by the interplay between nano-curcumin’s enhanced membrane permeability and black shallot’s antioxidant-immunomodulatory actions (Tran et al. 2023b; Hajimirzaei et al. 2025). Collectively, these findings support a synergistic, multi-targeted mechanism by which EBSC concurrently attenuates oxidative stress, nociceptive signaling, and the inflammatory cascade – mechanistically superior to single-component interventions (Hajimirzaei et al. 2025; El-Desoky et al. 2021).

Across all tested models, EBSC demonstrated significant improvements compared with the negative control, with measurable effects in both behavioral parameters (e.g., reaction latency, rectal temperature, and paw edema) and molecular markers (e.g., TRPV1, COX-2, PGE_2_, TNF-α, IL-6, and IL-10), all statistically validated through one-way ANOVA (*p*< 0.05, *n*= 8). The strong linear and sigmoidal regression fits confirm not only the potency but also the pharmacodynamic predictability of EBSC. These correlations justify dose optimization and support the formulation’s reproducibility in future translational investigations (Zhang et al. 2025). The inverse relationships between EBSC dose and key pro-inflammatory markers, alongside the positive correlation with IL-10, further reinforce its bidirectional immunomodulatory potential.

With its favorable safety margin (no adverse events up to 5.000 mg/kg) and consistent pharmacological effects, EBSC emerges as a promising phytopharmaceutical candidate (Rayan et al. 2020). Its multi-pronged activity – targeting pain, fever, and inflammation – makes it particularly suitable for integrative therapy in chronic inflammatory disorders, including hormone-related cancers, where conventional NSAIDs pose long-term safety concerns (Tran and Tran 2025). The synergistic combination of black shallot and nano-curcumin also addresses the bioavailability limitations associated with the individual agents, offering improved systemic distribution and enhanced efficacy. Given its low toxicity, broad therapeutic coverage, and scalable preparation, EBSC represents a viable candidate for development into standardized oral formulations or adjunct therapies, particularly in clinical contexts where resistance or intolerance to synthetic drugs is increasing.

Despite its promising profile, this study has limitations. Pharmacokinetic data for EBSC components were not assessed, yet such information is essential for determining systemic bioavailability and metabolic fate. The current work focused on acute models; therefore, chronic and disease-specific models (e.g., auto-immune arthritis and neuropathic pain) should be investigated to validate long-term efficacy. Additionally, to strengthen translational relevance, future studies should incorporate multiple time-point sampling, include appropriate placebo controls to distinguish vehicle effects, and apply the 3Rs (Replacement, Reduction, and Refinement) to optimize animal use and ensure ethical compliance.

## Conclusions

This study demonstrates the dose-dependent and synergistic therapeutic potential of EBSC in modulating nociceptive, febrile, and inflammatory responses. EBSC produced statistically significant effects across both behavioral and molecular endpoints (*p*< 0.05, *n* = 8), supported by strong dose-response correlations (*R^2^* > 0.98). With a favorable safety margin and a multi-targeted mechanism involving TRPV1, COX-2, PGE_2_, and cytokine modulation, EBSC represents a promising phytopharmaceutical candidate for the integrative management of inflammation-associated conditions. Future investigations will incorporate extended time-point analyses, placebo controls, and adherence to the 3Rs principles to enhance translational relevance and ethical robustness. Further development into standardized oral formulations is warranted to fully harness its therapeutic potential.
